# Analysis of the transgenerational iron deficiency stress memory in *Arabidopsis thaliana* plants

**DOI:** 10.3389/fpls.2015.00745

**Published:** 2015-09-17

**Authors:** Irene Murgia, Sonia Giacometti, Alma Balestrazzi, Stefania Paparella, Cristina Pagliano, Piero Morandini

**Affiliations:** ^1^Department of Biosciences, University of MilanoMilano, Italy; ^2^Department of Biology and Biotechnology ‘L. Spallanzani’, University of PaviaPavia, Italy; ^3^Applied Science and Technology Department – BioSolar Lab, Polytechnic University of TurinAlessandria, Italy

**Keywords:** *Arabidopsis thaliana*, chlorophyll, DNA strand breaks, Fe deficiency, photosynthetic apparatus, transgenerational memory, somatic homologous recombination, seed longevity

## Abstract

We investigated the existence of the transgenerational memory of iron (Fe) deficiency stress, in *Arabidopsis thaliana*. Plants were grown under Fe deficiency/sufficiency, and so were their offspring. The frequency of somatic homologous recombination (SHR) events, of DNA strand breaks as well as the expression of the transcription elongation factor *TFIIS-like* gene increase when plants are grown under Fe deficiency. However, SHR frequency, DNA strand break events, and *TFIIS-like* gene expression do not increase further when plants are grown for more than one generation under the same stress, and furthermore, they decrease back to control values within two succeeding generations grown under control conditions, regardless of the Fe deficiency stress history of the mother plants. Seedlings produced from plants grown under Fe deficiency evolve more oxygen than control seedlings, when grown under Fe sufficiency: however, this trait is not associated with any change in the protein profile of the photosynthetic apparatus and is not transmitted to more than one generation. Lastly, plants grown for multiple generations under Fe deficiency produce seeds with greater longevity: however, this trait is not inherited in offspring generations unexposed to stress. These findings suggest the existence of multiple-step control of mechanisms to prevent a genuine and stable transgenerational transmission of Fe deficiency stress memory, with the tightest control on DNA integrity.

## Introduction

“Priming” in plants can be defined as the intensification of defense responses occurring against previously encountered hostile factors, usually mediated by a complex network of priming targets and mechanisms (Pastor et al., 2013a; Balmer et al., 2015). Well-known examples of priming are the systemic acquired resistance (SAR; Fu and Dong, 2013) as well as the emission of volatile organic compounds (VOCs) upon plant attack by herbivores or by exposure to abiotic stress, which can trigger systemic defense responses even in neighboring plants (Aranega-Bou et al., 2014; Lee and Seo, 2014). Early responses induced by priming mechanisms include changes in redox homeostasis and production of specific reactive oxygen species (ROS) signals, interacting with various other signaling molecules, such as hormones and reactive nitrogen species (RNS; Molassiotis et al., 2010; Mukherjee et al., 2010; Mittler et al., 2011; Pastor et al., 2013a,b). Priming can last long after the first exposure to stress (Conrath et al., 2006) and long-lasting responses may be mediated by chromatin remodeling, such as histone modifications and DNA methylation (Jaskiewicz et al., 2011; Paszkowski and Grossniklaus, 2011; Pastor et al., 2013a; Vriet et al., 2015).

The inheritance of the primed state in the progeny is termed transgenerational memory and its occurrence, upon a challenge with abiotic stress (UV-C) or elicitors of plants defenses (flagellin), has been demonstrated using SHR (somatic homologous recombination) trap reporter lines. SHR events restore the marker gene β-glucuronidase (GUS), whose enzymatic activity is easily detected by the well-known GUS staining method (Tinland et al., 1994; Molinier et al., 2006; Puchta and Hohn, 2012). Various analyses of the stress memory inheritance upon exposure to various abiotic (nutritional, heat stress) (Verhoeven and van Gurp, 2012; Bilichak et al., 2015) as well as biotic stresses (Luna et al., 2012; Pieterse, 2012; Rasmann et al., 2012; Slaughter et al., 2012) have been recently reported.

However, other research groups have not confirmed the existence of a genuine stress memory in successive generations. The same *Arabidopsis thaliana* SHR-trap lines described by Molinier et al. (2006) have been exposed to an array of different abiotic stresses (e.g., salt, osmotic, freezing, oxidative, UV-B, UV-C), revealing only a sporadic transgenerational transmission of stress memory (Pecinka et al., 2009). The authors suggest that the observed transgenerational stress effects on SHR might be occurring in a stochastic manner, and might not belong to a general defense strategy against abiotic stresses (Pecinka et al., 2009), as also suggested in Boyko et al. (2010).

Resetting pathways, responsible for the “erasure” of stress memory and for protecting plants against negative effects of the accumulation of epigenetic modifications, have been proposed (Hauser et al., 2011; Paszkowski and Grossniklaus, 2011). Indeed, the mechanism of “erasure” of the vernalized state in subsequent generations recently led to the identification of ELF6, possessing H3K27me3 demethylase activity (trimethylation of histone H3 on lysine 27) as the protein responsible for the erasure of chromatin modifications regulating the floral repressor locus FLC (Crevillen et al., 2014).

Moreover, a screen for *Arabidopsis* mutants impaired in the erasure of epigenetic stress memory, identified the nucleosome remodeller Morpheus’ Molecule 1 MOM1 as a chromatin regulator acting in the restoration of the *Arabidopsis* epigenome to a pre-stress condition, together with another chromatin regulator Decrease in DNA Methylation 1 DDM1 (Iwasaki and Paszkowski, 2014). The analysis of loci activated by heat stress and still transgenerationally active in *ddm1mom1* double mutants progeny plants identified 340 genes, out of around a total 3000 genes activated by same stress conditions (Tittel-Elmer et al., 2010), suggesting that DDM1 and MOM1 control only a fraction of the erasure of the transgenerational stress memory (Iwasaki and Paszkowski, 2014).

Up to now, no studies on plant transgenerational stress memory occurring under Fe deficiency have been reported, and with the present work we therefore intend to fill such a gap in knowledge.

Iron is an essential element for plants, since it is involved in a wide range of housekeeping functions; iron is also involved in the response to biotic and abiotic stresses (Jeong and Guerinot, 2009; Ravet et al., 2009; Kieu et al., 2012; Ravet and Pilon, 2013; Briat et al., 2015). Research on the mechanisms by which plants acquire Fe from the soil through the roots and transport it to sink organs, without incurring the toxic effects of the free, redox-active form of Fe, has led to quite a complex view of regulation of Fe homeostasis and trafficking, from subcellular organelle to whole plant system (Kobayashi and Nishizawa, 2012; Vigani et al., 2013; Briat et al., 2015).

Plant growth and development are severely impaired when Fe uptake from the soil through the root apparatus is unable to satisfy the Fe demand of aerial parts and sink organs. Fe deficiency most frequently occurs when plants are grown in alkaline soils, where Fe solubility is reduced; such nutritional stress represents a severe burden for agriculture, in term of productivity and crop quality and, in turn, for human nutrition (Murgia et al., 2012, 2013; Briat et al., 2015).

In the present work we investigated the existence of the transgenerational Fe deficiency memory in *A. thaliana* plants. An SHR-trap line (Molinier et al., 2006; Puchta and Hohn, 2012) and wt control line (ecotype Col) were grown in various experimental conditions of Fe deficiency. The Fe deficiency memory was evaluated in subsequent generations, exposed or not to stress, by following DNA damage, SHR events, expression of transcription elongation factor *II-S* (*TFIIS* and *TFIIS-like*) genes as well as physiological parameters (chlorophyll content, O_2_ evolution, protein profile of the photosynthetic apparatus, seed longevity).

## Materials and Methods

### *Arabidopsis thaliana* Growth

*Arabidopsis thaliana* wt Col and SHR-trap 1445 line (background Col) were grown in control or alkaline soil, in a greenhouse at 21–25°C, 100 μE m^-2^ s^-1^, in long day conditions (16 h/8 h light/dark) unless otherwise specified, and watered with deionized water. Control soil was Technic n.1 DueEmme (Netherlands; pH 6.6) whereas alkaline soil (pH 7.7 or pH 8.4) was prepared by supplementing Technic n.1 soil with CaO (6 and 8 g CaO/kg soil, respectively). Soil was then moistened with water and thoroughly hand-mixed, its pH measured after few hours and adjusted with further supplements of CaO, as needed. Soil was then mixed again and its pH measured several times in the next 2 days and each time adjusted to the target pH with CaO, before use. At the end of the experiments, pH of alkaline soils was measured again; the observed decrease in pH (even after prolonged growth, as in case of plants grown till maturity for seed collection) was never higher than 0.2 pH units.

Sterilized *A. thaliana* seeds (45–60 seeds/plate) were placed on square Petri dishes (10 cm × 10 cm) containing AIS medium with either 50 μM final concentration Fe(III)-EDTA (indicated as control, +Fe; Murgia et al., 1998), or without Fe addition (indicated as -Fe).

### Seeds Collection, Maintenance, and Sterilization

Seeds were collected from mature brownish siliques and, after 1 week maintenance at room temperature, were either stored at -20°C (preserved seeds) or stored at room temperature for various time-lengths. Seeds were surface sterilized by fumigation with chlorine fumes; for that, seeds were placed in microcentrifuge tubes and placed open, in a desiccator jar. Immediately prior to sealing the jar, 3 ml of concentrated HCl were added to a solution of 15% bleach and seeds were kept inside the jar for 3–4 h.

### O_2_ Evolution and Chlorophyll Quantification

Oxygen evolution was measured with a Clark-type oxygen electrode (Hansatech, Ltd., King’s Lynn, Norfolk, UK) at 25°C by using a leaf disk electrode chamber. Seedlings (11 days-old, 30–50 mg each independent sample) were mounted in a sealed gas-tight chamber. Calibration was performed with 1 mL air at 25°C, 101.32 kPa containing 8.58 μmol O_2_. Prior to measurements, 200 μl bicarbonate buffer (1 M Na_2_CO_3_: 1 M NaHCO_3_: 1:9) was added in the chamber. Net oxygen rates were measured at two light intensities (100 and 800 μE m^-2^ s^-1^) and analyzed with OxylabV1.15 software. Seedlings were then removed from the chamber and put in a vial containing 1–3 ml dimethylformamide for chlorophyll extraction. Chlorophyll extraction and quantification was performed according to Tarantino et al. (2005).

### Comet Assay

For nuclei extraction, *A. thaliana* seedlings (14 days-old, 90 seedlings per independent sample, roughly corresponding to 100–200 mg) were frozen in liquid nitrogen, immediately chopped with a sharp blade and transferred into 400 μl of Chopping solution (PBS 1X, 10 mM EDTA, pH 7.0). The nuclei suspension was then filtered with a 50 μm mesh funnel (Sysmex Partec GmbH, Görlitz, Germany) and subsequently mixed with 300 μl of 1% (w/v) low melting point agarose (Sigma-Aldrich, Milan, Italy) in phosphate-buffered saline (PBS) buffer at 37°C. Two drops of the resulting suspension were then pipetted onto agarose precoated slides and solidified on ice. To let the DNA unwind for the detection of single strand breaks (SSBs) and double strand breaks (DSBs), slides were incubated for 20 min in the dark, at 4°C, in Alkaline Buffer (1 mM EDTA, 300 mM NaOH, pH 13.0). The slides were neutralized by washing in TBE buffer (89 mM Tris base, 89 mM Boric acid, 2 mM EDTA), and then electrophoresed in the same buffer for 5 min, 1 V cm^-1^, at 4°C in the dark. After electrophoresis, slides were washed in 70% (v/v) ethanol for 5 min, in ethanol 100% for 10 min and allowed to dry overnight at room temperature. Slides were stained with 20 μl of DAPI (4′,6-diamidino-2-phenylindole; 1 μg ml^-1^, Sigma-Aldrich; Menke et al., 2000). For each slide, 100 nucleoids were scored using a fluorescence microscope (Leica DM4000 B LED, Leica Microsystems GmbH, Wetzlar, Germany) with an excitation filter of 340–380 nm and a barrier filter of 400 nm. Nucleoids were classified and results were expressed in arbitrary units (a.u.) according to Collins (2004). For each treatment, three replicated samples were analyzed in two independent experiments.

### GUS Staining

β-Glucuronidase staining solution [1 mM sodium phosphate buffer pH 7.0, 10 mM EDTA, 0.3% Triton-X (v/v), 2 mM potassium ferrocyanide, 2 mM potassium ferricyanide, 0.5 mg/ml X-glucuronide] was infiltrated into submerged *A. thaliana* 2–3 weeks-old leaves (20–120 mg weight range of each sampled leaf) or 11 days-old seedlings by vacuum, for about 2 h. Samples were then incubated overnight at 37°C in the dark, rinsed in distilled water and destained 1 h in acetic acid/ethanol solution (3:1 v/v) and a final rinse in 70% (v/v) ethanol. SHR events, revealed as blue sectors, were evaluated and counted under a stereomicroscope (Leica).

### Quantitative Real-Time Polymerase Chain Reaction (qRT-PCR)

RNA was extracted from seedlings (after 14 days from germination) with TRIzol^®^ reagent (Life Technologies, Carlsbad, CA, USA), by following manufacturer’s instructions. qRT-PCR was carried out using the iTaq^TM^ universal SYBR^®^ Green one-step kit (Bio-Rad Laboratories, Hercules, CA, USA) using a Rotor-Gene 6000 PCR apparatus (Corbett Robotics Pty Ltd., Brisbane, QLD, Australia). Reaction conditions were as follows: reverse transcription at 50°C for 10 min, denaturation at 95°C for 1 min, and 40 cycles of 95°C 15 s and 60°C 30 s. After the amplification a melting reaction was carried out. The expression profiles of *A. thaliana TFIIS* gene (TAIR annotation At2g38560) and *TFIIS-like* gene (TAIR annotation At5g09850; Macovei et al., 2011) were obtained by using β-Tubulin (TAIR annotation At1g20010) and Ubiquitin4 (TAIR annotation At5g20620) as reference genes. Primers were designed with the Primer Design online tool Primer3^1^ and checked with an OligoAnalyzer tool (Integrated DNA Technologies^2^). For each oligonucleotide set, a no-template water control was used. qRT-PCR data analysis was carried out according to Vandesompele et al. (2002). Statistical analysis was performed with SigmaStat (Systat Software, Richmond, CA, USA).

### Thylakoid Isolation

*Arabidopsis thaliana* seedlings (11 days-old, around 200–600 mg each independent sample) were frozen in liquid nitrogen and ground to obtain a fine powder, which was further homogenized in 50 mM HEPES-NaOH, pH 7.2, 5 mM MgCl_2_, 10 mM NaCl, and 0.5 M sucrose. The homogenate was filtered through six layers of cotton cloth, then membranes were pelleted by centrifugation for 10 min at 3,000 *g* at 4°C. Pellets were resuspended in previous buffer devoid of sucrose and spun down for 10 min at 4,500 g. Finally, thylakoids were resuspended in 50 mM HEPES-NaOH, pH 7.2, 5 mM MgCl_2_, 10 mM NaCl, and 0.1 M sucrose. The chlorophyll concentration was determined spectrophotometrically after extraction in 80% (v/v) acetone according to Arnon (1949).

### Gel Electrophoresis and Western Blotting

Sodium dodecylsulfate polyacrylamide gel electrophoresis (SDS-PAGE) was performed on a 12.5% (w/v) polyacrylamide gel containing 5 M urea using Laemmli’s system (Laemmli, 1970); pre-stained protein markers (Precision plus, Bio-Rad) were used for the estimation of apparent molecular mass of the main thylakoid proteins. After electrophoretic separation, proteins were either detected by using a silver staining protocol, as described in Shevchenko et al. (1996), or transferred onto a nitro-cellulose membrane. Further immunodetection was performed with specific antisera against PsaA, CP43, PsbO, D1, PsbE (Agrisera codes AS06172, AS111787, AS05092, AS05084, AS06112, respectively) and LHCII polypeptides, by using the alkaline phosphatase conjugate method, with 5-bromo-4-chloro-3-indolyl phosphate/nitro blue tetrazolium as chromogenic substrates (Sigma-Aldrich).

### Correlation and Statistical Analysis

Correlation analysis was performed as described in Menges et al. (2008) and Berri et al. (2009), both in the linear and in the log space. Statistical analysis was performed by using Student’s *t*-test.

## Results

### *A. thaliana* Plants Grown under Fe Deficiency Show Enhanced SHR Frequency with a Subsequent Decrease within Two Generations Grown under Fe Sufficiency

*Arabidopsis thaliana* wt Col plants (control generation c0) were grown in either of two alkaline soils, at either pH 7.7 or pH 8.4. The collected seeds represent the stressed progeny generation 1 (“pH 7.7 s1” and “pH 8.4 s1,” respectively).

**FIGURE 1:**
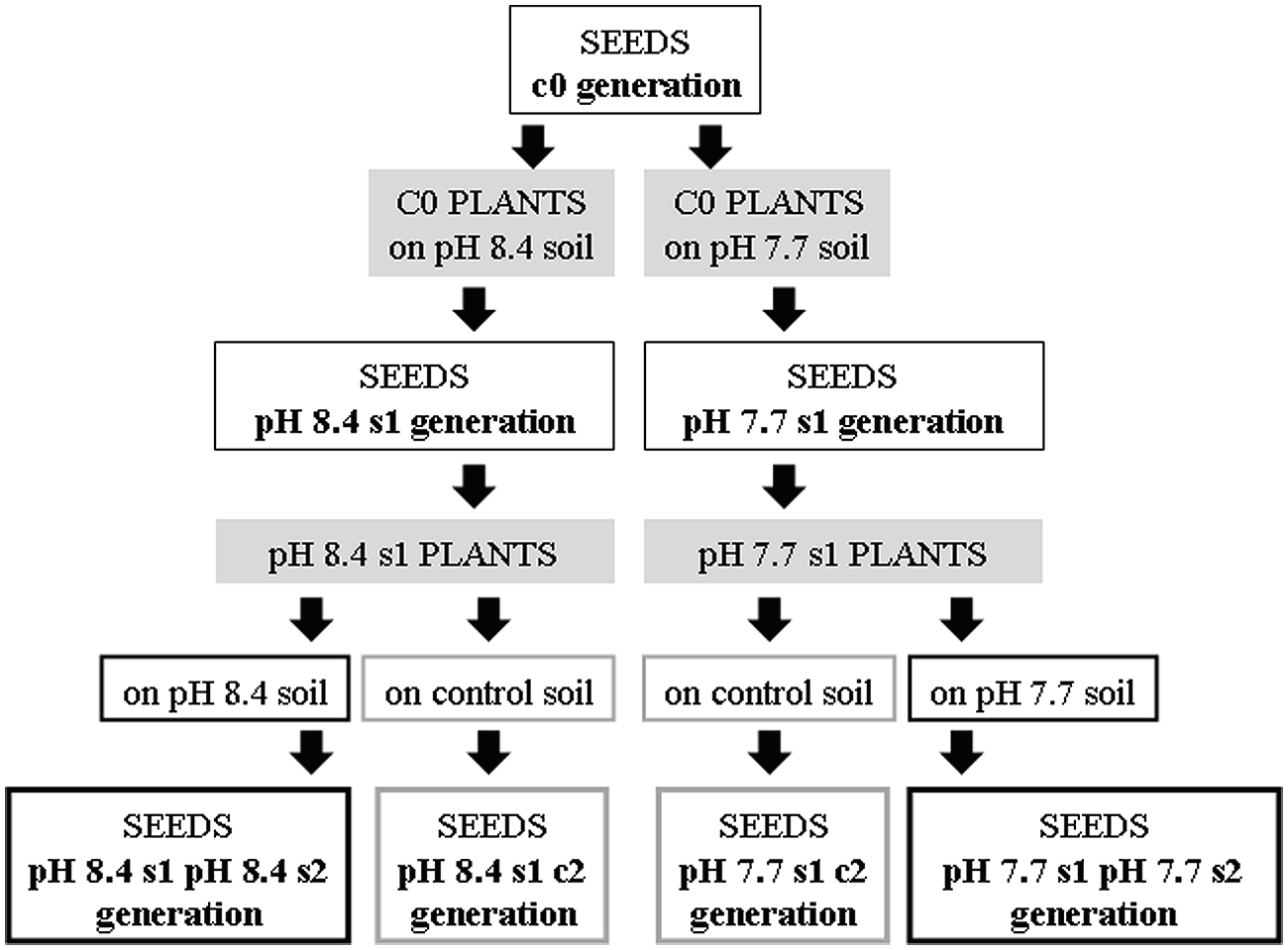
**Production of the *Arabidopsis thaliana* plants with generational exposure to Fe deficiency.** *A. thaliana* wt ecotype Col or *A. thaliana* SHR-trap 1445 line (background Col) were used as control c0 generation (c0 seeds). c0 seeds grown in control soil produced seeds that are still named c0; c0 seeds grown in either pH 7.7 or pH 8.4 soil produced pH 7.7 s1 or pH 8.4 s1 seeds, respectively. pH 7.7 s1 seeds grown in either pH 7.7 or control soil produced pH 7.7 s1 pH 7.7 s2 or pH 7.7 s1 c2 seeds, respectively. pH 8.4 s1 seeds grown in either pH 8.4 or control soil produced pH 8.4 s1 pH 8.4 s2 or pH 8.4 s1 c2 seeds, respectively.

Col pH 7.7 s1 and pH 8.4 s1 plants, when grown either in control, pH 7.7 (Supplementary Figures S1A,B) or pH 8.4 soil (Supplementary Figure S1C), show higher biomass production than corresponding c0 plants grown in the same conditions (Supplementary Figure S1), thus suggesting the existence of a memory of Fe deficiency stress in the offspring. To confirm the existence of a genuine transgenerational memory, the SHR-trap line 1445 (control generation c0), was grown in either of two alkaline soils, at either pH 7.7 or 8.4, with production of pH 7.7 s1 and pH 8.4 s1 generation seeds, respectively (**Figure 1**).

Both wt Col and SHR-trap line 1445 pH 7.7 s1 and pH 8.4 s1 generations were then grown in control, pH 7.7 or pH 8.4 soil. The resulting generation 2 seeds were named pH 7.7 s1 pH 7.7 s2 and pH 8.4 s1 pH 8.4 s2 respectively, whereas seeds produced from an s1 generation grown in control soil were named pH 7.7 s1 c2 and pH 8.4 s1 c2, respectively. Progeny seeds from c0 plants grown in control soil were named c0 seeds. A scheme summarizing the different steps in the production of the plant lines and the relative nomenclature, is shown in **Figure 1**.

**FIGURE 2:**
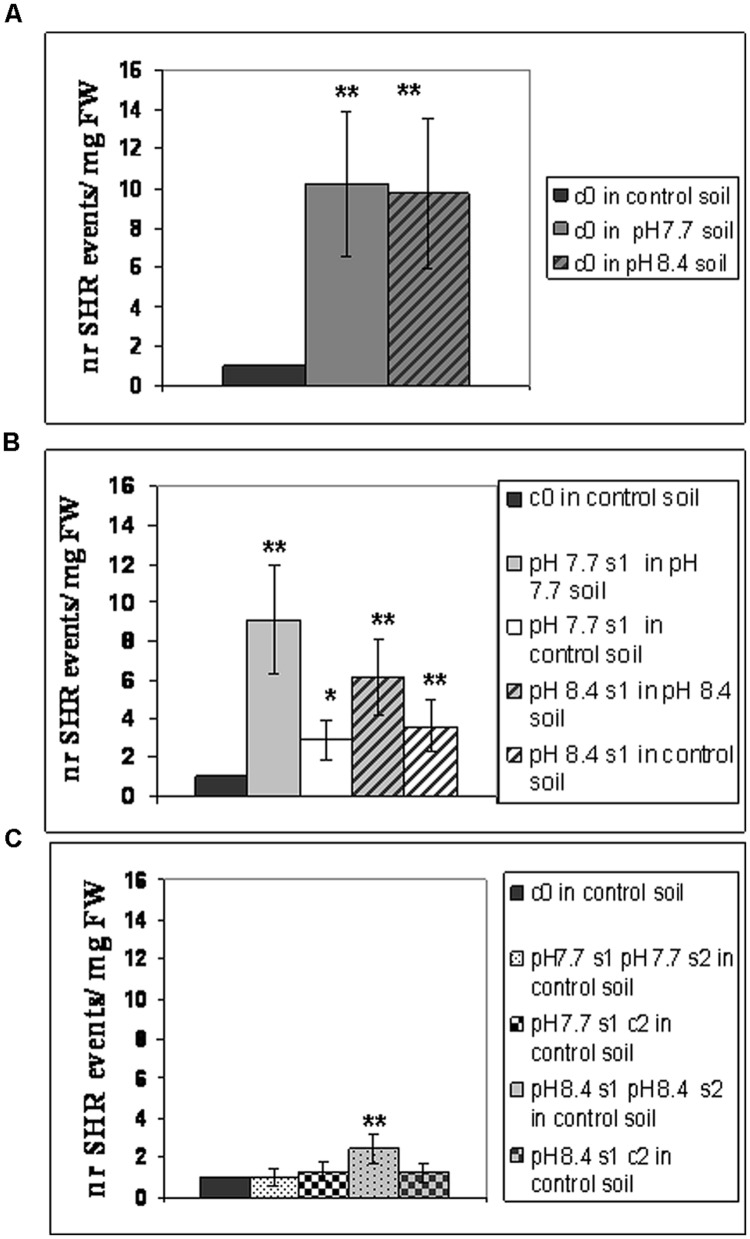
**Somatic homologous recombination (SHR) in *A. thaliana* plants with generational exposure to Fe deficiency and grown under Fe deficiency or sufficiency. (A)** *A. thaliana* SHR-trap 1445 c0 plants grown in control, pH 7.7 or pH 8.4 soil. **(B)** s1 plants (pH 7.7 s1 and pH 8.4 s1) grown in control, pH 7.7 or pH 8.4 soil. **(C)** s1s2 and s1c2 plants (pH 7.7 s1 pH 7.7 s2, pH 8.4 s1 pH 8.4 s2, pH 7.7 s1 c2, pH 8.4 s1 c2) grown in control soil. For all experiments, plants were grown for 2–3 weeks and rosette leaves were GUS-stained for detection of SHR events, counted as number of independent blue spots/mg fresh weight. Each value represents the mean spots number ± SE in five to twenty leaves. Significant differences (with respect to control c0 value) are indicated with ^∗∗^(*p* < 0.01) or ^∗^(*p* < 0.05), according to Student’s *t*-test.

Frequency of SHR events was then evaluated in the following SHR-trap 1445 generations: c0 (**Figure 2A**), s1 (**Figure 2B**), s1s2 and s1c2 (**Figure 2C**); for that, they were grown in control, pH 7.7 or pH 8.4 soil followed by GUS staining of leaves. SHR frequency in c0 plants grown at either pH 7.7 or pH 8.4 is roughly ten times higher than that observed in c0 plants grown in control soil (**Figure 2A**). Such higher SHR frequency was maintained, though it did not increase further in plants grown under Fe deficiency and produced from mother plants which were also grown under the same stress, as shown for pH 7.7 s1 grown in pH 7.7 soil and pH 8.4 s1 grown in pH 8.4 soil (**Figure 2B**). The lack of additive effect suggests a control of SHR frequency, with a plateau effect. Notably, when either pH 7.7 s1 or pH 8.4 s1 are grown in control soil, the SHR frequency was drastically reduced (**Figure 2B**) but was still higher than control, suggesting occurrence of a faint “stress memory.” Such memory was, however, completely erased when plants were grown for two successive generations in control soil, as was evident in pH 7.7 s1 c2 and pH 8.4 s1 c2 generations grown again in control soil (**Figure 2C**). It is also interesting to note that growth for two successive generations under Fe deficiency did not reinforce stress memory in offspring unexposed to stress, as observed in pH 7.7 s1 pH 7.7 s2 and pH 8.4 s1 pH 8.4 s2 lines, grown in control soil (**Figure 2C**).

Taken together, these results show that the number of SHR events increased in plants grown under Fe deficiency, both in mild or in severe alkaline soil and that frequency of SHR events returned to control values within two generations unexposed to stress. Moreover, growth of more than one generation in alkaline soil was not associated with a higher frequency of SHR events.

**FIGURE 3:**
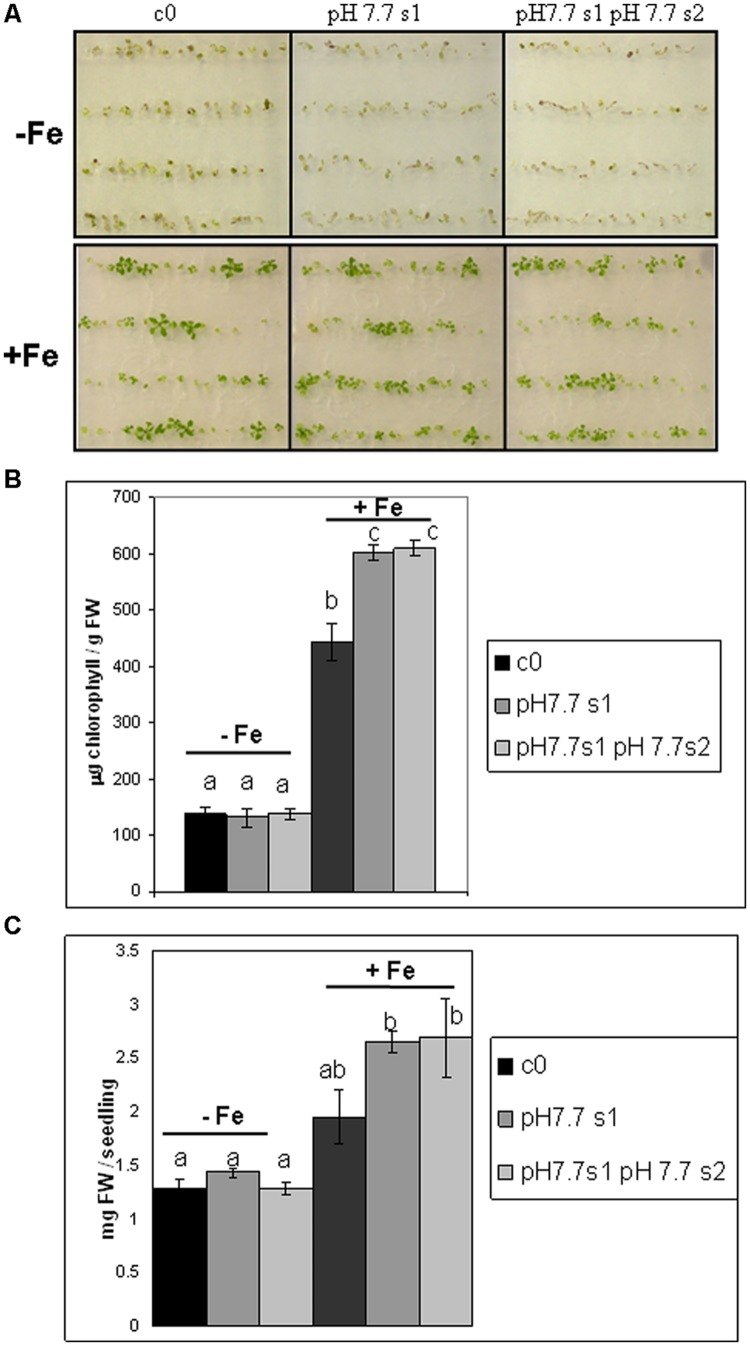
***Arabidopsis thaliana* seedlings with generational exposure to Fe deficiency and grown under Fe deficiency or sufficiency. (A)** *A. thaliana* SHR-trap 1445 seedlings, from either control generation (c0) or from single (pH 7.7 s1) or multiple generational exposure to Fe deficiency (pH 7.7 s1 pH 7.7 s2) germinated for 11 days in control AIS medium (+Fe), or AIS medium without Fe (-Fe). **(B)** Chlorophyll content, expressed as mg chlorophyll/g fresh weight of seedlings described in **(A)**. **(C)** Weight (expressed as mg fresh weight/seedling) of seedlings described in **(A)**. Bars represent mean values ± SE, from at least three biological samples consisting of twenty seedlings each. Significant differences are indicated with letters (*p* < 0.05), according to Student’s *t-*test.

**FIGURE 4:**
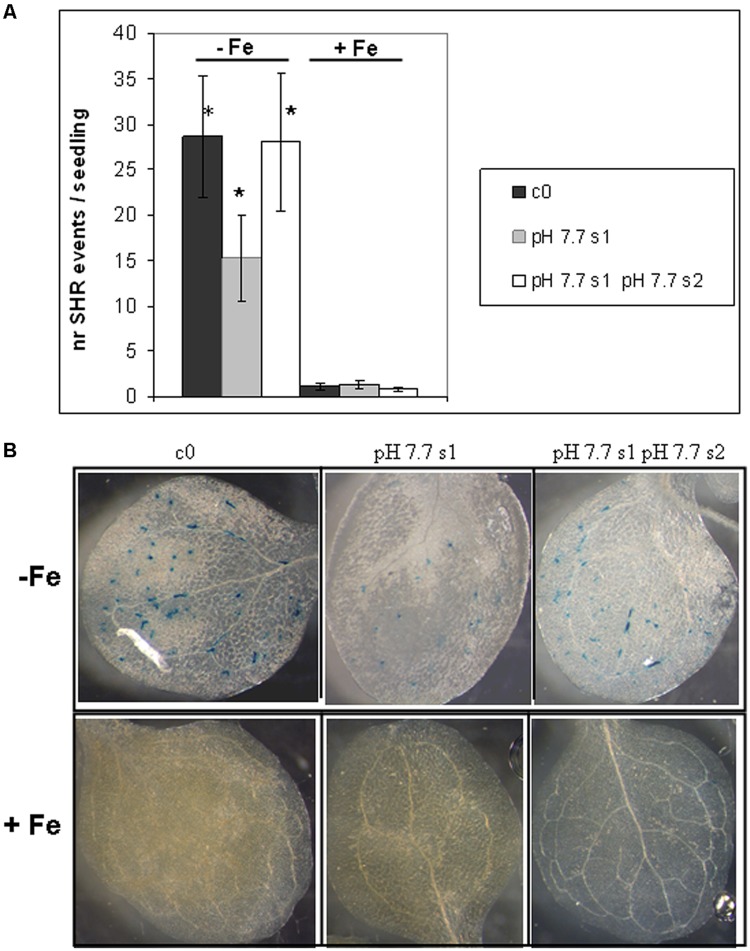
**Somatic homologous recombination events in *A. thaliana* seedlings with generational exposure to Fe deficiency and grown under Fe deficiency or sufficiency. (A)** SHR events in *A. thaliana* SHR-trap 1445 seedlings, from control generation (c0), from single (pH 7.7 s1) or multiple generational exposure to Fe deficiency (pH 7.7 s1 pH 7.7 s2) grown for 11 days in control AIS medium (+Fe), or in AIS medium without Fe (-Fe). Seedlings were stained for GUS expression and SHR events evaluated as number of spots/seedling. Values are the mean spot number ± SE in ten seedlings. Significant differences (with respect to control c0) are indicated with ^∗^(*p* < 0.05), according to Student’s *t*-test. **(B)** Representative seedlings, described in **(A)**, stained for detection of GUS activity.

To reduce any variability of results resulting from stunted growth occurring in alkaline soil, SHR frequency was also measured in c0, pH 7.7 s1 and pH 7.7 s1 pH 7.7 s2 seedlings, when germinated on control AIS medium (+Fe) or in AIS medium without iron supplement (-Fe) (**Figure 3A**). All tested seedlings growing under Fe deficiency were equally affected, in terms of chlorophyll content (**Figure 3B**). Both pH 7.7 s1 and pH 7.7 s1 pH 7.7 s2 seedlings, grown in +Fe, showed instead a significantly higher chlorophyll content (but not significantly higher fresh weight), with respect to their control c0 seedlings in the same condition (**Figures 3B,C**). As already observed for SHR frequency (**Figure 2**), chlorophyll content and seedlings fresh weight did not further increase upon exposure to Fe deficiency for multiple generations (**Figures 3B,C**). SHR frequency was measured in seedlings treated as described in **Figure 3**; results confirm that Fe deficiency strongly enhanced SHR frequency, as observed for SHR-trap seedlings c0, pH 7.7 s1, pH 7.7 s1 pH 7.7 s2 grown under Fe deficiency (**Figure 4**), thus confirming the observations about plants grown on alkaline soil (**Figure 2**).

As also observed with plants grown in soil, SHR frequency was not enhanced further in seedlings exposed for more than one generation to Fe deficiency stress (**Figure 4A**), suggesting again the plateau effect mentioned above. Vice-versa, unstressed offspring seedlings of stress-treated plants, had an SHR frequency similar to c0, when grown in control soil (**Figure 4A**).

These results show that the number of SHR events increased in seedlings grown under Fe deprivation and that the frequency of SHR events returned to control values within one generation grown under Fe sufficiency. Moreover, growth of more than one generation under Fe deprivation was not associated with a higher frequency of SHR events, thus confirming observations with plants grown in alkaline soil.

### DNA Damage Profiles and Expression of the *TFIIS-like* Gene are Enhanced in *A. thaliana* Seedlings Grown under Fe Deficiency but Neither in s1 nor in s1s2 Generations Grown under Fe Sufficiency

Genotoxic stress in plants is generally regarded as a main factor which affects genome stability, impairing productivity (Balestrazzi et al., 2011; Waterworth et al., 2011). Since the genotoxic impact of Fe deficiency is still unexplored in plants, an investigation was carried out to assess the DNA damage profiles occurring under Fe deficiency, using the Alkaline SCGE (single cell gel electrophoresis, or Comet assay), which measures both SSBs and DSBs.

**FIGURE 5:**
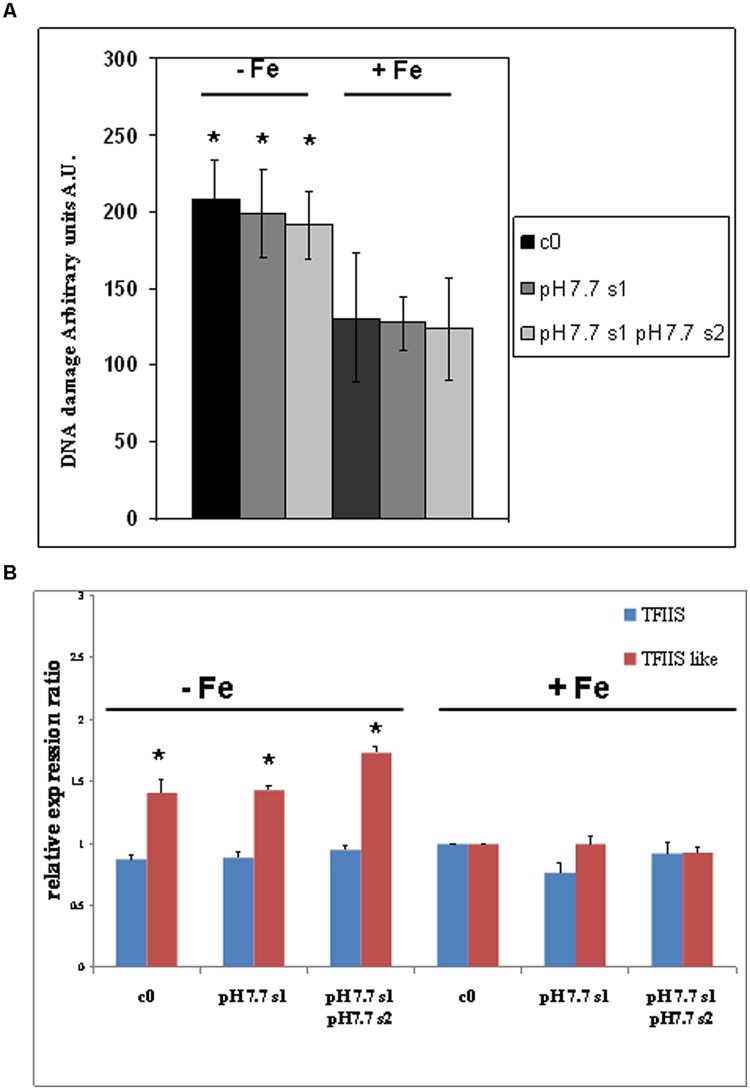
**DNA damage and expression of *AtTFIIS* and *AtTFIIS-like* genes, in *A. thaliana* seedlings with generational exposure to Fe deficiency and grown in control AIS medium (+Fe) or AIS medium without Fe (-Fe). (A)** Alkaline Comet assay on 14 days-old SHR-trap 1445 c0, pH 7.7 s1, pH 7.7 s1 pH 7.7 s2 seedlings, when germinated in control (+Fe) or in -Fe AIS medium. One hundred cells were scored for each sample. Values are expressed as mean ± SD of three replicates from two independent experiments. **(B)** Expression profiles of *AtTFIIS* (in blue) and *AtTFIIS-like* genes (in red) in the samples described in **(A)**, by qRT-PCR analysis. Values are the result of three independent experiments and have been normalized to the expression value in control c0 grown in +Fe. Statistically significant differences (with respect to control c0) are indicated with ^∗^(*p* < 0.05), according to Student’s *t*-test.

DNA lesions were quantified in nuclei isolated from SHR-trap 1445 c0, pH 7.7 s1, pH 7.7 s1 pH 7.7 s2 seedlings, when germinated in control (+Fe) or in -Fe medium. Control c0 seedlings grown in +Fe showed a DNA damage value of 130.6 ± 41.7 a.u., which can be attributed to the experimental manipulations of nuclei; such a value is similar to those quantified in both pH 7.7 s1 seedlings (127.4 ± 17.5 a.u.) and pH 7.7 s1 pH 7.7 s2 seedlings (123.6 ± 33.2 a.u.), when in +Fe (**Figure 5A**). Vice-versa, as already observed for the SHR events, the extent of DNA damage increased in c0 seedlings (208.3 ± 25.3 a.u.), in pH 7.7 s1 seedlings (198.8 ± 28.4 a.u.) and in pH 7.7 s1 pH7.7 s2 seedlings (191.0 ± 22.1 a.u.), under Fe deficiency (-Fe; **Figure 5A**). Moreover, in accordance with the SHR events, growth for more than one generation under Fe deficiency did not further increase DNA damage, as such values measured in -Fe pH 7.7 s1 and -Fe pH 7.7 s1 pH 7.7 s2 seedlings were similar (**Figure 5A**).

These results show that DNA damage increased in seedlings grown under Fe deprivation and that it returned to control values within one generation grown under Fe sufficiency. Moreover, growth of more than one generation under Fe deprivation was not associated with a more pronounced DNA damage, as also observed for SHR events.

Mechanisms involved in genome protection include nucleotide excision repair (NER; Fousteri and Mullenders, 2008), which operates through the TC (transcript-coupled) sub-pathway to remove DNA lesions and block RNA polymerase II and restore transcription. One of the components of TC-NER pathway is the transcription elongation factor TFIIS, which associates with RNA polymerase II, possibly enabling repair under UV-C exposure (Lagerwerf et al., 2011).

Plants possess the *TFIIS* gene (At2g38560/RDO2) which is involved in the regulation of seed dormancy and development, as described for *Arabidopsis* by Grasser et al. (2009) and Mortensen and Grasser (2014). Also, *TFIIS-like* genes have been identified and characterized in *Medicago truncatula* (Macovei et al., 2011) and rice (*Oryza sativa* L.) (Macovei et al., 2014); such genes encode proteins sharing some common features with the canonical TFIIS proteins. Both genes are responsive to genotoxic stress induced by x-ray and salinity stress in seedlings and mature plants (Macovei et al., 2014). A gene with 60% similarity to *MtTFIIS-like*, according to Phytozome (v10.2^3^, Goodstein et al., 2012), is present in the *A. thaliana* genome, namely At5g09850 gene, which we therefore named *AtTFIIS-like*. Alignment of protein sequences of *AtTFIIS-like* and *MtTFIIS-like* (Medtr3g095380, according to Phytozome) is shown in Supplementary Figure S2 where the TFIIS domain is highlighted. Both TFIIS-like proteins share the LW motif, required to accomplish the nuclear import of proteins (Ling et al., 2006).

The expression profiles of both *AtTFIIS* and *AtTFIIS-like* genes were studied by qRT-PCR in SHR-trap 1445 c0, pH 7.7 s1, pH7.7 s1 pH 7.7 s2 seedlings germinated in control (+Fe) or in -Fe. As shown in **Figure 5B**, *TFIIS* expression was not dependent either on the Fe nutritional status, or on the stress pedigree of the tested lines, being similar in all tested seedlings, when grown in +Fe as well as in -Fe (**Figure 5B**). On the other hand, the expression of the *TFIIS-like* gene significantly increased in all seedlings (c0, pH 7.7 s1, pH 7.7 s1 pH 7.7 s2) grown in -Fe, compared with what was observed in +Fe (**Figure 5B**).

These results show that expression of the *AtTFIIS-like* gene increased in seedlings grown under Fe deprivation and that such expression returned to control values within one generation grown under Fe sufficiency. Moreover, growth of more than one generation under Fe deprivation was not associated with an increased expression of the *AtTFIIS-like* gene.

Correlation analysis of gene expression has been successfully applied for identifying new candidate genes for a given biological process (Beekwilder et al., 2008; Murgia et al., 2011). Such analysis, was performed on At2g38560 (*TFIIS/RDO2*), At5g09850 (*TFIIS-like*), At1g01210, At2g13640, At2g27780, At3g25940, At3g48060, At4g07950, At4g18720, At4g24200, At5g05140, At5g27310 and At5g51360, i.e., all the *A. thaliana* genes, which are annotated as *TFIIS* or similar, according to the *Arabidopsis* Information resource (TAIR^4^), and detected by the ATH1 Affymetrix microarray gene chip. However, the analysis did not detect any strong positive correlation between any of the tested genes and the genes involved in the Fe-deficiency response (e.g., FRO1, FIT1, IRT1, Popeye, etc.) with a threshold for the Pearson’s coefficient set to 0.6 (data not shown).

AtTFIIS-like has been identified as one of the putative proteins forming subunit 26 of the Mediator complex, and named AtMed26_3 (Mathur et al., 2011); interestingly, Mediator subunit 16 gene (At4g04920), which is also part of the Mediator complex representing a bridge between RNA polymerase and specific transcriptional activators, functions in the regulation of iron homeostasis (Yang et al., 2014; Zhang et al., 2014). Correlation analysis of MED16 did not reveal a strong correlation with any of the Fe deficiency response genes whose expression is MED16-dependent and reported in Zhang et al. (2014) (data not shown). Results of such correlation analyses, together with the evidence that MED16 exerts a profound impact on the Fe deficiency response without changing its expression under Fe deprivation (Zhang et al., 2014), suggests that the alteration of *TFIIS-like* gene expression, under Fe deficiency, even if not striking, may be of relevance for the Fe deficiency response.

### Photosynthetic Parameters in s1, s1s2, and s1c2 Generations: Oxygen Evolution, Chlorophyll Content, and Protein Composition of the Photosynthetic Apparatus

**FIGURE 6:**
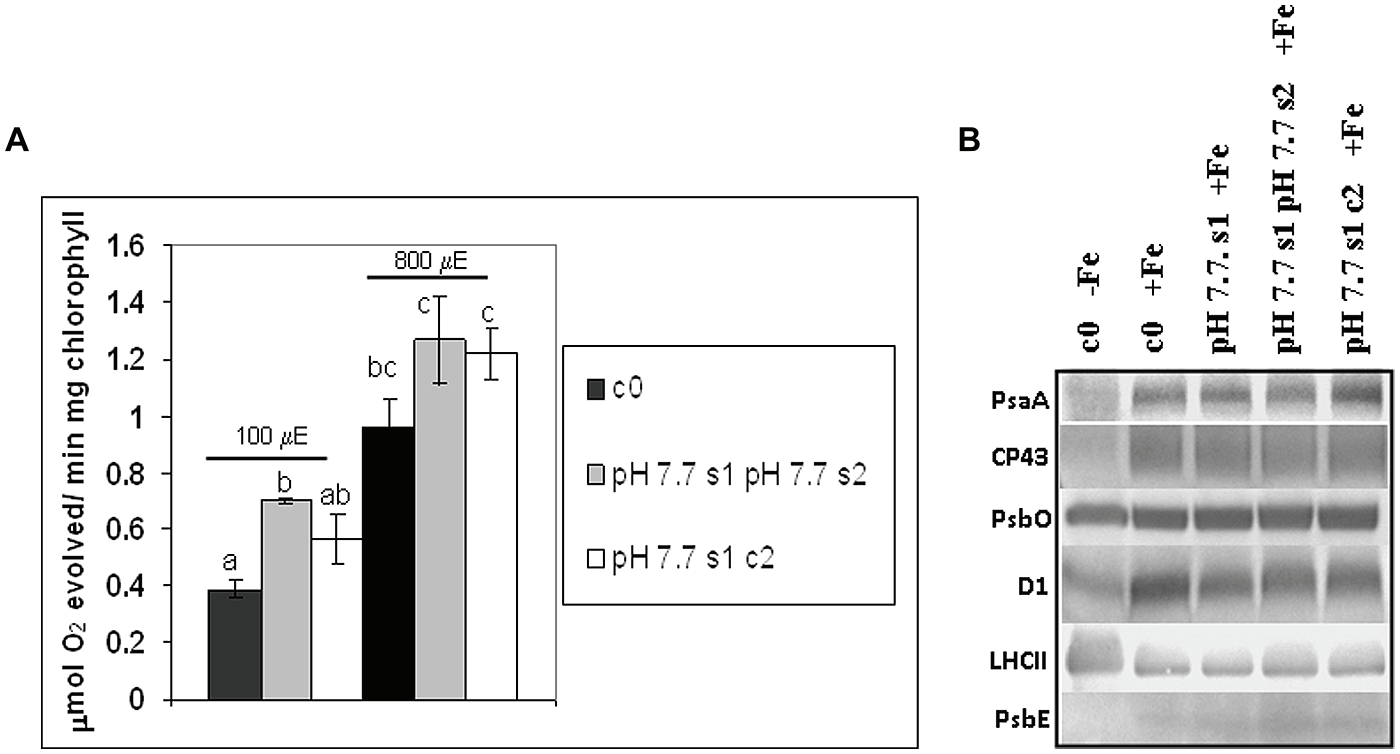
**O_2_ evolution and protein profile of photosynthetic apparatus in *A. thaliana* seedlings with generational exposure to Fe deficiency. (A)** SHR-trap 1445 c0, pH 7.7 s1 pH 7.7 s2, pH 7.7 s1 c2 seedlings were grown for 11 days in control AIS medium and net O_2_ evolution (expressed as μmol O_2_ evolved min^-1^ mg chlorophyll^-1^) was measured under illumination at either 100 or 800 μE m^-2^ s^-1^. Values are mean ± SE of three biological replicas, each consisting of at least 15 seedlings. Letters represent statistical differences, according to Student’s *t*-test, with *p* < 0.05. **(B)** Western blot analysis with antibodies against PsaA, CP43, PsbO, D1, LHCII, and PsbE of thylakoid membranes purified from 11 days-old SHR-trap 1445 c0 seedlings germinated in -Fe AIS medium or from 11 days-old SHR-trap 1445 c0, pH 7.7 s1, pH 7.7 s1 pH 7.7 s2, pH 7.7 s1 c2 seedlings germinated in control (+Fe) AIS medium. Samples corresponding to 1 μg chlorophyll were loaded on each lane.

O_2_ evolution was higher in pH 7.7 s1 pH 7.7 s2 SHR-trap line seedlings than in control c0, when both were grown in +Fe, thus suggesting that the photosynthetic apparatus is more efficient in seedlings whose parental plants experienced Fe deficiency (**Figure 6A**), in agreement with what was observed for chlorophyll content. Interestingly, O_2_ evolution values were not significantly higher in pH 7.7 s1 c2 than in control c0 (**Figure 6A**), indicating a loss of this trait in the offspring unexposed to stress. When seedlings are illuminated with light intensities well above growth light, i.e., at 800 μE m^-2^ s^-1^ (**Figure 6A**), the higher O_2_ evolution in pH 7.7 s1 pH 7.7 s2 is no longer statistically significant (**Figure 6A**).

These results show that O_2_ evolution was higher in seedlings grown in control conditions and with parental plants which had experienced Fe deficiency, in agreement with what was observed for chlorophyll content; however, this trait was lost within two generations unexposed to stress.

As a result of Fe deficiency, changes in the protein profiles of different plant parts and compartments, among which are the thylakoid membranes, have been reported (Andaluz et al., 2006; López-Millán et al., 2013). Within the thylakoid membranes, both photosystem I (PSI) and photosystem II (PSII) are affected by Fe deficiency; however, it can be expected that there will be a more severe effect on PSI than on PSII, due to its higher number of Fe atoms per photosystem: 14 Fe atoms/PSI *versus* 2 Fe atoms/PSII (Yadavalli et al., 2012; Briat et al., 2015).

To further explore the impact of Fe deficiency stress and the existence of the memory of such stress on the protein composition of the photosynthetic apparatus, the reaction center subunits of PSI, PsaA, and of PSII, D1, together with other PSII polypeptides, among which the inner antenna protein CP43, the outer antenna protein LHCII, the extrinsic polypeptide of the Oxygen Evolving Complex PsbO and the PsbE protein (a subunit of cytochrome b_559_ binding an heme cofactor), were analyzed by SDS-PAGE and western blotting, in c0 seedlings germinated in -Fe, as well as in c0, pH 7.7 s1, pH 7.7 s1 pH 7.7 s2, pH 7.7 s1 c2 seedlings when germinated in +Fe (Supplementary Figure S3 and **Figure 6B**). Upon equal loading of chlorophyll in the protein gels for all analyzed samples, the amount of PsaA, as well as that of CP43 and D1, was dramatically reduced in -Fe c0 seedlings, when compared with +Fe c0 seedlings (**Figure 6B**). Such results are in agreement with proteomic profiles observed in thylakoids isolated from Fe-deficient *Beta vulgaris* leaves (Andaluz et al., 2006). However, it should be noted that such a decrease in PsaA has not been observed in Fe-deficient rice seedlings (Yadavalli et al., 2012). Fe deficiency dramatically affects the level of cytochrome b_559_ in -Fe c0 seedlings, indeed its a subunit, PsbE, could not be detected in -Fe c0 seedlings (**Figure 6B**). Notably, under Fe deficiency, no decrease was observed in the amount of the PsbO subunit in any of the tested samples, thus suggesting that, at least in *A. thaliana*, Fe deficiency does not equally affect all the protein components of the two photosystems. Lastly and conversely to the behavior of the reaction center proteins of PSI (PsaA) and PSII (D1), and the inner antenna protein of PSII (CP43), the amount of the peripheral antenna proteins LHCII was not reduced in -Fe c0 seedlings when compared to any other +Fe samples.

A lack of substantial alterations in the protein profile of photosynthetic proteins in any of the generations grown in +Fe, i.e., pH 7.7 s1, pH 7.7 s1 pH 7.7 s2 and in pH 7.7 s1 c2, when compared to +Fe c0 seedlings (**Figure 6B**), could be observed.

To further investigate chlorophyll as a hallmark of growth of the mother plant under Fe deficiency, the various c0, s1, s1s2, s1c2 generations of wt Col, described in **Figure 1**, were also tested by growing seedlings in control (+Fe) or -Fe medium. As already observed for SHR-trap line (**Figure 3B**), chlorophyll content was higher in pH 7.7 s1, pH 7.7 s1 pH 7.7 s2, pH 8.4 s1 and pH 8.4 s1 pH 8.4 s2 Col seedlings, when compared to c0 Col seedlings in same condition (Supplementary Figure S4). Interestingly, chlorophyll concentration was higher in pH 7.7 s1 c2 and pH 8.4 s1 c2 offspring grown in control conditions than c0 itself (Supplementary Figure S4), indicating that this trait can be maintained for more than one offspring generation unexposed to stress. Also, the plateau effect observed for SHR events (**Figures 2** and **4**), DNA damage (**Figure 5A**), expression of TFIIS-like (**Figure 5B**) is confirmed once more, since chlorophyll content was statistically similar in pH 7.7 s1 and pH 7.7 s1 pH 7.7 s2 seedlings, as well as in pH 8.4 s1 and pH 8.4 s1 pH 8.4 s2 seedlings, when grown in control conditions (Supplementary Figure S4).

The various wt Col generations described above were also germinated in -Fe; as expected (see also **Figure 3B**) a reduction in chlorophyll content was observed in -Fe seedlings when compared to control ones (Supplementary Figure S4). The tested s1, s1s2, and s1c2 seedlings showed chlorophyll content of variable intensity, with respect to c0, when in -Fe (Supplementary Figure S4). Such an effect was not observed in the SHR-trap line (**Figure 3**), where all tested seedlings, when in -Fe, showed the same chlorophyll content, regardless of stress pedigree.

### Seed Longevity Increases in s1s2 Seeds but not in s1c2 Ones

Seed longevity can be severely affected due to oxidative damage occurring during prolonged storage and/or exposure to unfavorable environmental conditions (Donà et al., 2013). As a consequence of aging, seed viability is compromised (Kranner et al., 2010). In order to assess the effects of Fe deficiency on seed quality, germination of after-ripened (stored at room temperature) or of dormancy-preserved (stored at -20°C) Col c0, s1, s1s2, s1c2 generations was assessed; for that, seed batches of various ages were used (3 months, 26–30 months, and 6 years-old seeds). As positive controls, after-ripened 26–30 months-old c0 seeds of the transgenic *A. thaliana* lines tAPX OE 14.2 and tAPX OE 8.5 overexpressing thylakoidal ascorbate peroxidase, which show an enhanced resistance to oxidative stress (Murgia et al., 2004) were also used; these lines allowed to investigate plant responses to various abiotic and biotic oxidative stresses (Murgia et al., 2004; Yao and Greenberg, 2006; Laloi et al., 2007; Skirycz et al., 2011). Nomenclature of all the tested seeds that keeps track of stress history of the mother plants is according to **Figure 1**.

Germination curves at 25°C in the light were assessed over a 6-days time span, as a proxy for seed longevity; for that, seeds were first cold-stratified (4 days at 4°C in the dark) to break any residual dormancy and to promote synchronous germination.

**FIGURE 7:**
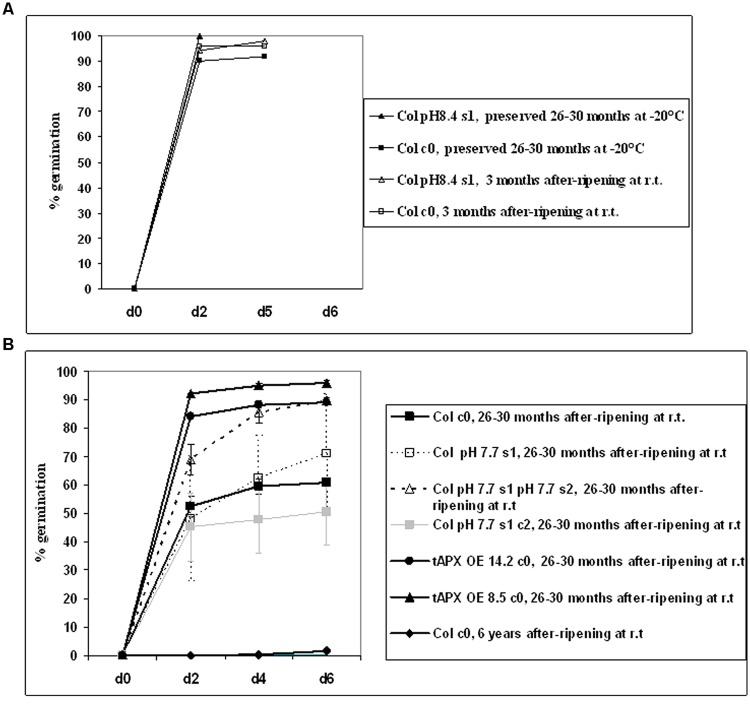
**Germination of *A. thaliana* seeds, produced from plants with transgenerational exposure to Fe deficiency. (A)** Germination of c0 or pH 8.4 s1 Col seeds, after-ripened at room temperature for 3 months, or preserved at -20°C for 26–30 months. **(B)** Germination of c0, pH 7.7 s1, pH 7.7 s1 pH 7.7 s2, pH 7.7 s1 c2 Col seeds, after-ripened for 26–30 months at room temperature. As controls, c0 14.2 tAPX OE, 8.5 tAPX OE seeds after ripened for 26–30 months at room temperature as well as c0 Col seeds after-ripened for 6 years at room temperature were also used. Values correspond to mean percent germination ± SE from at least three plates containing 100 seeds each.

As expected, young (3 months-old) after-ripened c0 or pH 8.4 s1 Col seeds all germinated within 2 days; full germination was also observed in older (26–30 months-old) c0 seeds, if stored at -20°C (**Figure 7A**). Next, germination of c0, pH 7.7 s1, pH 7.7 s1 pH 7.7 s2, pH 7.7 s1 c2 after-ripened 26–30 months Col seeds was measured. Germination of c0 Col seeds was below 50% (**Figure 7B**) and therefore much lower than that of same-age seeds stored at -20°C (**Figure 7A**). Germination of pH 7.7 s1 pH 7.7 s2 Col seeds was much higher, being above 80% (**Figure 7B**), whereas germination curves similar to control were observed in pH 7.7 s1 or pH 7.7 s1 c2 Col seeds (**Figure 7B**). Unfortunately, lack of 26–30 months after-ripened pH 7.7 s1 pH 7.7 s2 c3 Col seeds prevented us from further exploring longevity traits. Lastly, 26–30 months after-ripened c0 14.2 and 8.5 tAPX OE seeds (used as positive controls) showed 90–100% germination (**Figure 7B**), as high as that observed in young (3 months-old) after-ripened wt Col seeds, whereas fairly old c0 Col seeds (6 years after ripening) did not germinate (**Figure 7B**).

These results showed that growth of more than one generation under Fe deficiency has a positive impact on the longevity of seeds produced.

## Discussion

The analysis of stress-induced plant priming and related transgenerational stress memory have been intensively debated due to their great potential for crop improvement (Hauser et al., 2011; Vriet et al., 2015). Transgenerational memory could be of advantage for subsequent generations exposed to nutritional stress: indeed, in the open, some environmental abiotic stresses such as cold, drought, etc. might not even occur during offspring growth, due to fluctuations of environmental conditions; even pathogen attacks might be limited in time. In such cases, the advantage of “erasing” any memory of stress that means all the burden of epigenetic modification, might outbalance the advantage of maintaining the memory of that stress in the progeny. Vice-versa, nutritional deficiencies due to soil composition are chronic stresses that hardly change with time and therefore, in such a case, the maintenance of the burden of epigenetic modifications induced by nutritional stresses might be of advantage for plants whose seeds often fall in close proximity to the mother plant. Despite the increase in recent years of reports on transgenerational stress memory, including the effects of nutrients limitations (Kou et al., 2011; Verhoeven and van Gurp, 2012), up to now, the stable inheritance of Fe deficiency stress memory in offspring generations has not been analyzed.

We investigated, for the first time, whether a genuine transgenerational memory of Fe deficiency stress occurs in the model plant *A. thaliana*; we demonstrated that Fe deficiency alters DNA damage and repair and we analyzed such traits in unstressed offspring (Vriet et al., 2015); moreover, we also analyzed physiological effects of Fe deficiency occurring in unstressed offspring (Verhoeven and van Gurp, 2012), such as the status of the photosynthetic apparatus (chlorophyll content, O_2_ evolution, protein profile) and also seed longevity; all these traits are indeed agronomically and economically relevant.

Most importantly, we analyzed such traits not only in the first generation but also in the second one, with and without stress, to distinguish genuine transgenerational memory from other possible mechanisms, such as transmission, from mother to progeny plant, of enzymes, toxins, hormones, etc. which can be observed in progeny but do not represent genuine memory (Paszkowski and Grossniklaus, 2011; Verhoeven and van Gurp, 2012).

We showed that frequency of DNA damage and repair, evaluated through events of SHR and DNA strand breaks, increased in plants grown under various conditions of Fe deficiency (mild or severe alkaline soil, depletion of Fe from medium); however, their frequencies returned to control values within one or two offspring generations, as was also observed for SHR induced by various stresses and described in Pecinka et al. (2009). It was interesting that SHR frequency, in s1 or s2 generations unexposed to stress, immediately declined to control levels in experiments conducted in controlled medium, whereas it took one more generation in experiments conducted in soil; such lasting “stress memory” in soil, at least for one unstressed generation, but not in medium, suggests that growth in alkaline soil might trigger a more complex response to stress than the Fe deficiency response obtained by simple Fe deprivation. Nevertheless, the need to obtain sufficient amounts of seeds from plants exposed to Fe deficiency stress and with different stress pedigrees, together with the fact that soil alkalinisation is indeed the most common cause of Fe deficiency encountered by plants in the open, prevented us from choosing to carry out *in vitro* experiments only. Anyway, the data obtained from mild or severe alkaline pH (pH 7.7 or pH 8.4) confirmed the consistency of results obtained with soil-grown plants.

We also analyzed some photosynthetic parameters: chlorophyll content, O_2_ evolution, protein composition of photosynthetic apparatus. Chlorophyll content was still altered in two offspring generations unexposed to stress; however, erasure of such alteration in further generations might be supposed. Indeed, results of increased chlorophyll content, in offspring of plants grown under Fe deficiency and described in the present work, were occasionally not reproducible, as already reported for other transgenerational phenotypic effects: as an example, apomictic dandelion (*Taraxacum officinale*, common dandelion) grown under nutrients limitation, shows increased root:shoot ratio in offspring produced from plants grown under the same nutritional stress compared with the offspring from plants grown under control conditions: however, such a phenotype was variable and could not be always reproduced within the same lab and from the same authors (Verhoeven and van Gurp, 2012). As already pointed out by these authors, such lack of full consistency among various experiments, as far as chlorophyll content is concerned, could be due to subtle differences among experimental conditions which might override parental effects (Verhoeven and van Gurp, 2012).

Moreover, pH 7.7 s1 c2 seedlings, when germinated in control conditions, evolved less oxygen than pH 7.7 s1 pH 7.7 s2 seedlings in the same conditions. Provided that the chlorophyll content could be further tested in the offspring of the various generations already described in the present work, all the photosynthetic parameters measured so far do not support the existence of a genuine and reproducible transgenerational transmission of memory. The same observations apply to the increased weight, not always observed in s1 progeny grown in control conditions, as reported in the present work; also in such a case, subtle differences among experimental conditions which might override parental effects.

*Arabidopsis thaliana* AtTFIIS-like is one of the putative proteins of subunit 26 of the Mediator complex, i.e., MED26_3 (Mathur et al., 2011); although its expression is only slightly induced by Fe deficiency, nevertheless the expression of MED16, another subunit of Mediator which is undoubtedly involved in the regulation of the Fe deficiency response (Yang et al., 2014; Zhang et al., 2014), does not change at all under Fe deficiency, making the observed change in *AtTFIIS-like* gene expression, profoundly relevant. The reported data, highlighting the involvement of the *TFIIS-like* gene in the plant response to Fe-deficiency stress, provide for the first time evidence of the role played in this context by the TC-NER sub-pathway. The latter is critical for cell survival due to its anti-mutagenic properties, which protect DNA against the long-term effects of genotoxic stress (Fousteri and Mullenders, 2008). TC-NER, which specifically acts on the transcribed regions of the genome, thus preserving gene expression, has been previously demonstrated to participate in the response to gamma irradiation (Macovei et al., 2014), as well as heavy metals and osmotic stresses (Macovei et al., 2011). The present work contributes to further expand the range of stresses that trigger TC-NER, but also significantly contributes to the current scientific discussions on the putative link between transgenerational memory and DNA repair response in plants.

Longevity of seeds represents a research focus of several groups, due to the tremendous ecological, economic and nutritional impact that such physiological processes have (Devaiah et al., 2007) and indeed one of the most important questions facing plant science research is what determines seed longevity and dormancy (Grierson et al., 2011). The observed phenotype of increased longevity in offspring seeds of Fe deficiency stressed mother plants is new in the nutritional field and it also encourages further investigation in order to clarify the link between seed longevity, Fe content, its localization and mobilization as well as levels of different ROS species, in seeds produced from mother plants grown under Fe deficiency.

The next step in the elucidation of the Fe-deficiency associated traits described in the present work will be to understand if and how their molecular regulation is associated with epigenetic modifications.

## Conclusion

We described for the first time profiles of SHR events and of DNA damage, occurring under Fe deficiency in *A. thaliana* and we also identified a new Fe-deficiency responsive gene, i.e., At*TFIIS-like*. We then analyzed SHR, DNA damage, *AtTFIIS-like* gene expression, as well as a set of physiological parameters in a multiple-generation cohort of plants with different stress pedigrees. The data obtained suggest the existence of multiple-step control of mechanisms orchestrating the prevention of a genuine and stable transgenerational transmission of Fe deficiency stress memory, with the tightest control on DNA integrity. The production of a wider cohort of s3 (and successive) plant generations and the analysis of traits such as chlorophyll content, O_2_ evolution and in particular seed longevity in such extended cohort, when grown either in presence or absence of stress, will allow to further test this hypothesis.

## Author Contributions

IM conceived the work and wrote the manuscript; IM, SG, AB, SP, CP, and PM contributed the data; IM, SG, and PM contributed to the analysis and the discussion of data.

## Conflict of Interest Statement

The authors declare that the research was conducted in the absence of any commercial or financial relationships that could be construed as a potential conflict of interest.

## Footnotes

1 http://primer3.ut.ee

2 https://eu.idtdna.com/calc/analyzer

3 http://phytozome.jgi.doe.gov/pz/portal.html

4 https://www.arabidopsis.org/
