# Supplementary figures and images for: Analysis of the transgenerational iron deficiency stress memory in Arabidopsis thaliana plants
Source: Front Plant Sci. 2015 Sep 17;6:745. doi: 10.3389/fpls.2015.00745 (PMC4585125; doi:10.3389/fpls.2015.00745)

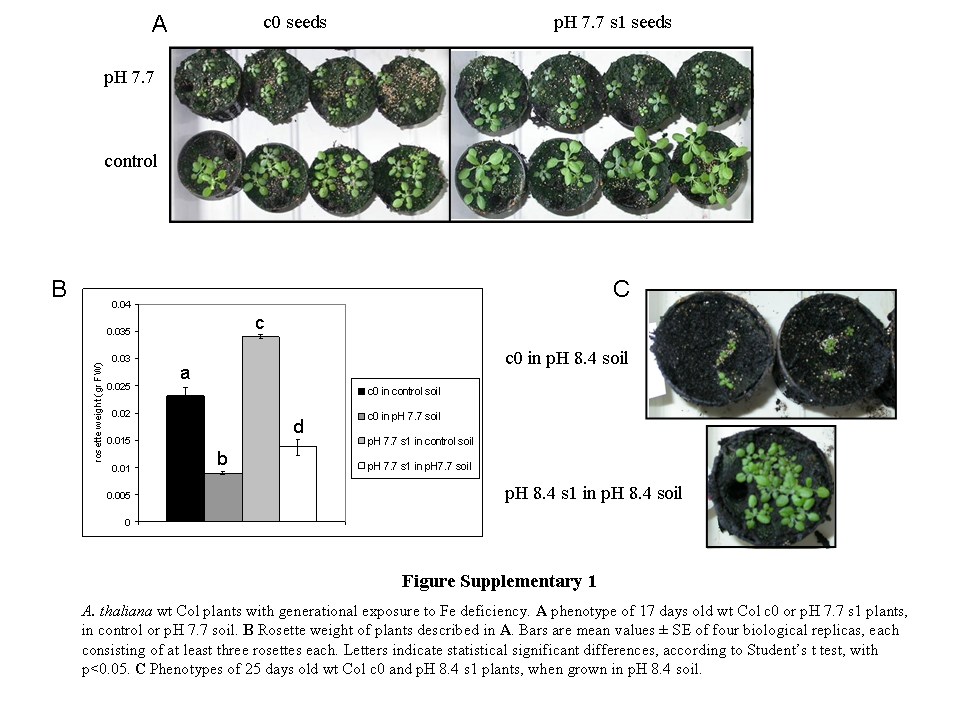

Supplement: Supplementary file 1 [file Image_1.TIF]

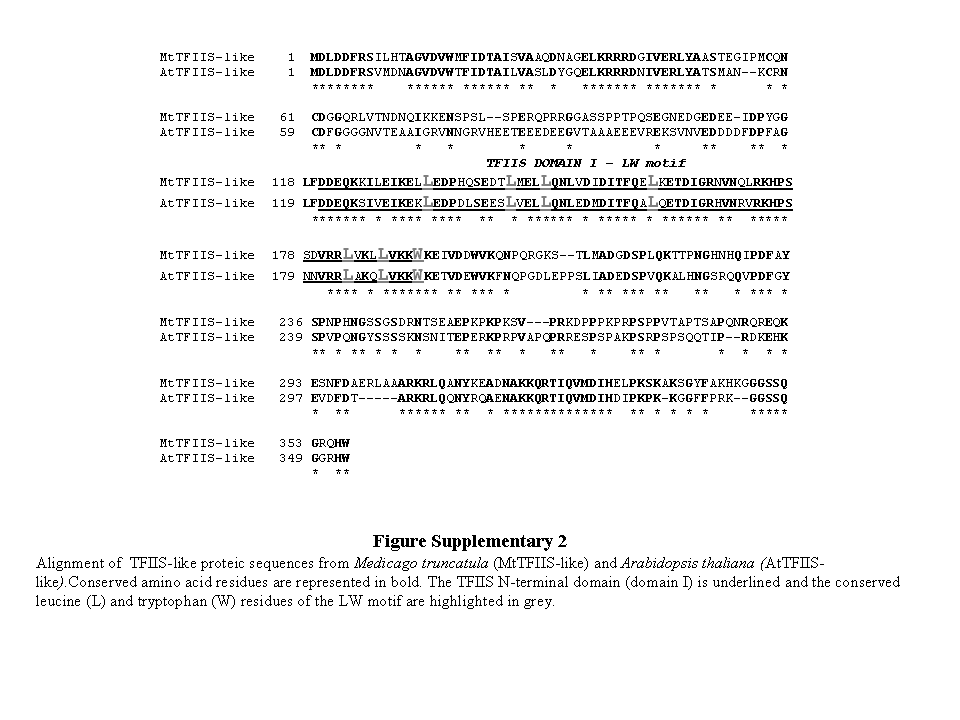

Supplement: Supplementary file 2 [file Image_2.TIF]

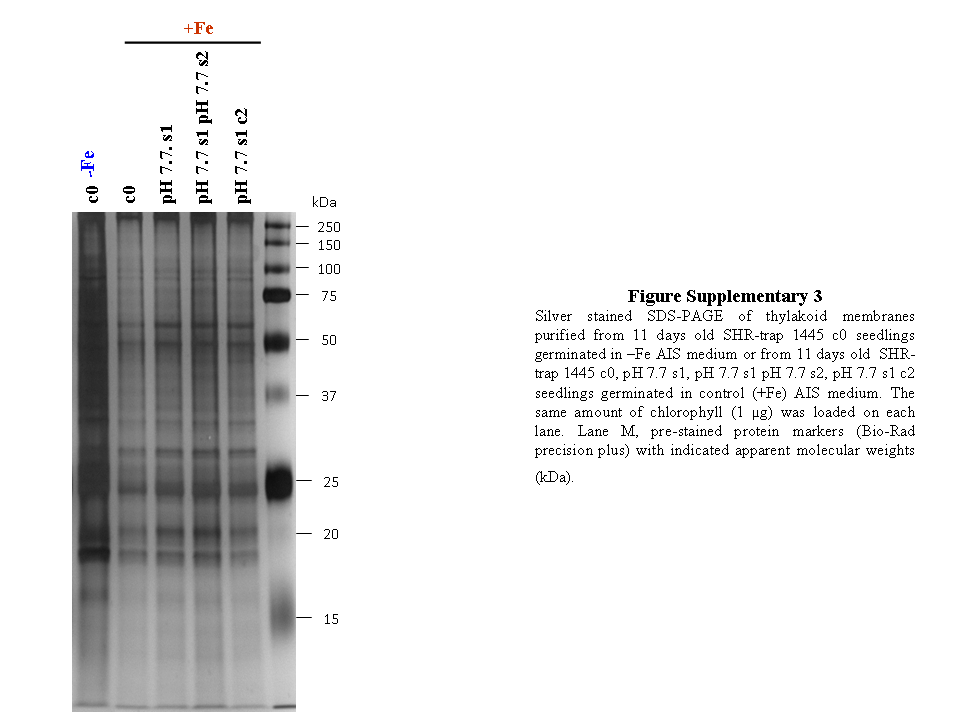

Supplement: Supplementary file 3 [file Image_3.TIF]

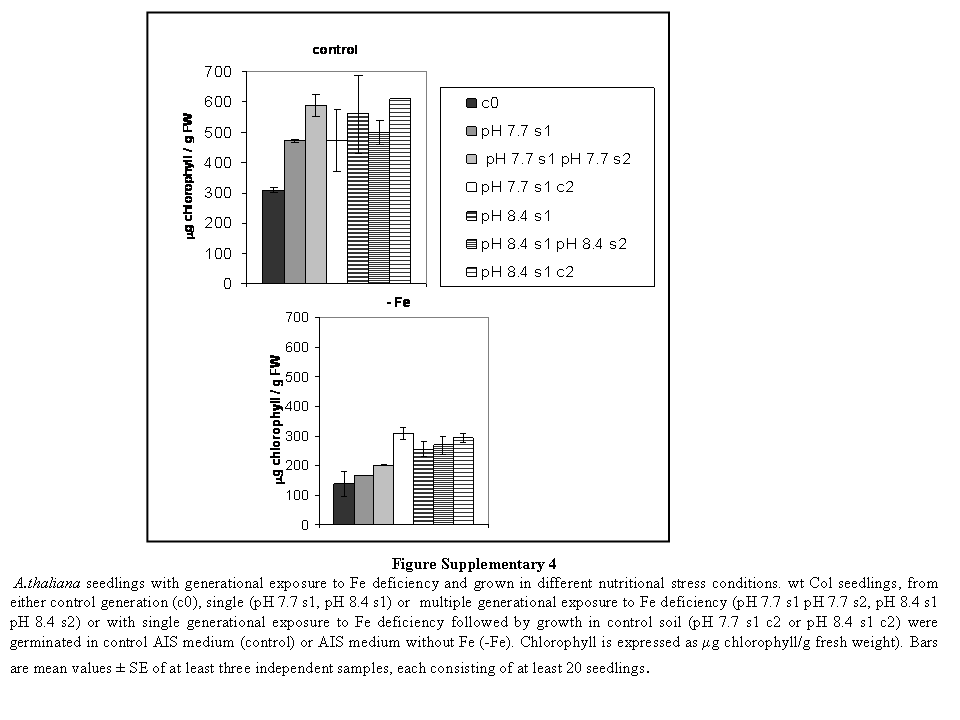

Supplement: Supplementary file 4 [file Image_4.TIF]
